# Social attitudes toward Tongqi among the general public and associated determinants: a mixed-methods study in Hubei Province, China

**DOI:** 10.3389/fpsyt.2025.1700396

**Published:** 2025-11-21

**Authors:** Fen Yang, Yufei Qiu, Yake Yue, Mengjie Tong, Jiali Liu, Lijuan Zeng, Juan Gu, Yiqing Yu

**Affiliations:** 1 College of Nursing, Hubei University of Chinese Medicine, Wuhan, Hubei, China; 2 Department of Nursing, Hubei Provincial Hospital of Traditional Chinese Medicine, Wuhan, Hubei, China; 3 Hubei Shizhen Laboratory, Wuhan, Hubei, China

**Keywords:** Tongqi, wives of gay men, men who have sex with men, social attitudes, China

## Abstract

**Background:**

In China, “Tongqi” (wives of men who have sex with men) remain a socially marginalized group. Public awareness and understanding of Tongqi are limited. Misconceptions and stigma persist, potentially hindering their access to social support and equitable treatment. However, empirical research in China on the general public’s attitudes toward Tongqi and the factors shaping these views remains scarce. This study aimed to investigate the general public’s social attitudes toward Tongqi and the factors influencing these attitudes.

**Methods:**

An explanatory sequential mixed-methods design was employed in Hubei Province, China, in 2024. The quantitative phase involved a structured survey of 587 participants, while the qualitative phase included in-depth interviews with 20 individuals from diverse social backgrounds. Quantitative data were analyzed using descriptive statistics, independent samples *t*-tests, one-way ANOVA and multiple linear regression. Qualitative data were thematically analyzed using NVivo 14.

**Results:**

The mean score of social attitudes toward Tongqi was 89.93 (SD = 16.60), indicating a moderately neutral stance. Social attitude scores were significantly associated with gender (*β* = 5.39, *P* < 0.001), education (*β* = 3.37, *P* < 0.05), marital status (*β* = -3.45, *P* < 0.001), occupational status (*β* = 3.99, *P* < 0.05), perceptions of the current social environment (*β* = -4.83, *P* < 0.001) and sexual orientation (*β* = 5.66, *P* < 0.01). Qualitative analysis revealed three key themes: (1) Cognitive aspect—awareness shaped by traditional and societal norms; (2) Affective aspect—public empathy and emotional orientation toward Tongqi; (3) Behavior aspect—expressed needs and public expectations for support mechanisms.

**Conclusion:**

The general public holds moderately neutral social attitudes toward Tongqi, influenced by multiple factors. Deep-rooted stigma, driven by conservative views on marriage and sexuality, continues to marginalize this group. Although emotional sympathy exists, the absence of public visibility and structural support limits meaningful social action. Future research should expand to diverse populations and sociocultural settings to inform targeted interventions. Multisectoral engagement is urgently needed to enhance awareness, reduce stigma, and establish comprehensive support systems for Tongqi.

## 1 Introduction

The phenomenon of Tongqi, referring to heterosexual women who are married to gay men, has emerged as a distinctive and complex social issue in China, attracting increasing public and scholarly attention in recent years (1). The term Tongqi is a neologism with unique cultural characteristics in China (2). In many Western societies, the Tongqi phenomenon is relatively uncommon due to widespread societal recognition of homosexuality, the legalization of same-sex marriage, and a strong cultural emphasis on individual autonomy and freedom (3). Conversely, the Tongqi phenomenon is more prevalent in China, shaped by a confluence of factors including traditional cultural norms, deeply rooted family values, and relatively inadequate legal protection mechanisms (4–6). China is home to the largest homosexual population globally (7). Studies indicate that approximately 90.0% of gay men in China enter heterosexual marriages, primarily to conceal their sexual orientation, avoid social stigma, and fulfill familial expectations such as producing offspring (2). By integrating data from the United Nations Women with the latest results of China’s national population census, the current number of Tongqi in China is estimated to range from 23 to 32 million (8). This represents a significant increase from the approximate 16 million reported over a decade ago, highlighting the growing scale of this social phenomenon.

Despite the continuous growth of the Tongqi population in China, prevailing public attitudes remain largely discriminatory and stigmatizing, posing significant barriers to their pursuit of social support and equitable treatment. Importantly, most of what is known about these social attitudes derives not from systematic surveys of the general public, but from Tongqi’s own accounts of their lived experiences. Cognitively, public awareness of Tongqi is limited and distorted by patriarchal traditions and heteronormative norms (7, 9), with many perceiving them as “queer”, “opportunistic”, “mentally unstable”, or even as “damaged” or “spoiled” goods and potential AIDS carriers (4, 6). These cognitive biases are also shaped by China’s underdeveloped and inconsistent sex education system. Influenced by Confucian cultural norms, sexual topics are often treated as private or taboo, leading parents, teachers, and students to avoid discussions in formal educational settings (10). Most primary and secondary school teachers lack systematic training and scientifically grounded teaching methods, while adolescents rely primarily on online sources and social media for sexual knowledge, where information is often inaccurate or incomplete (11, 12). Such misconceptions are further reinforced by the media’s preference for eye-catching headlines, coupled with a scarcity of research focusing on Tongqi (2, 4). Affectively, Tongqi are frequently confronted with opposition, ridicule, and blame from their communities, while expressions of empathy or compassion remain fragmented and rare (6, 13–15). Behaviorally, such negative stereotypes and affective orientations manifest in daily interactions, ranging from verbal insults and silent stares to harassment, bullying, and even physical violence, often without any social intervention or protection (6, 9, 16). Additionally, the absence of institutional backing in China, given that same-sex marriage remains illegal and relevant legal protections are underdeveloped, further compounds their marginalization (2, 5). These findings suggest that public attitudes toward Tongqi not only shape Tongqi’s immediate experiences of stigma and exclusion in social interactions but also influence structural conditions, including public discourse, policy priorities, and the distribution of social resources. Therefore, it is essential to investigate the general public’s social attitudes toward Tongqi and the factors underpinning them, to achieve a more comprehensive understanding of their marginalization and to identify potential entry points for social change.

Research on Tongqi in China remains relatively nascent and limited in scope (2, 4, 5). Existing studies have primarily concentrated on domains such as medicine, sociology, and law, addressing issues including marital survival, psychological distress, legal rights protection, social adaptation, and HIV-related health risks (2, 5, 17, 18). Methodologically, most investigations have relied on case interviews and small-scale qualitative designs, emphasizing the personal narratives and lived experiences of Tongqi. While these studies provide valuable insights into the dilemmas and vulnerabilities faced by this group, they remain constrained by their predominant reliance on Tongqi’s self-reported experiences. In particular, there has been little systematic exploration of the general public’s social attitudes toward Tongqi (19), although the fact that such attitudes play a critical role in shaping their social environment, access to support, and opportunities for empowerment.

This gap in the literature highlights the need for empirical studies that examine public perceptions and their determinants, which constitute the central focus of the present research. Addressing this gap, this study investigates the social attitudes toward Tongqi among the general public in Hubei Province, China. By employing a mixed-methods design that combines quantitative and qualitative approaches, this study aims to provide a comprehensive understanding of public attitudes and the factors influencing their formation. The findings are expected to offer empirical evidence for enhancing social awareness, promoting inclusion, and protecting the legitimate rights and interests of Tongqi. Furthermore, this study seeks to contribute to the academic discourse by offering new perspectives and directions for future interdisciplinary research in the fields of gender, sexuality, and social justice in contemporary Chinese society.

## 2 Methods

### 2.1 Study design

This study employed a sequential explanatory mixed-methods design, structured in two phases (20). The first phase involved quantitative research to assess social attitudes toward Tongqi and to identify key influencing factors. The second phase consisted of semi-structured interviews to complement and expand upon the quantitative findings, thereby offering deeper contextual insights into societal perceptions of Tongqi. By integrating both approaches concurrently and iteratively, this study strengthens the overall validity, depth, and interpretive strength of the findings, achieving a level of understanding that would be unattainable through a single-method approach (21).

### 2.2 Quantitative phase

#### 2.2.1 Participants

Participants were recruited using a convenience sampling method. The inclusion criteria were as follows: (1) aged 18 years or older; (2) possessing basic literacy skills and no communication barriers; and (3) providing informed consent and voluntary participation in this study. Setting the minimum age at 18 ensured that all participants were legally adults and likely to have more developed cognitive and social reasoning abilities, which are essential for providing valid responses.

#### 2.2.2 Data collection

The quantitative phase was conducted from June to August 2024 in Hubei Province, China. Data were collected online using the Wenjuanxing (Questionnaire Star) platform. The electronic questionnaire was disseminated through major social media platforms, including QQ, WeChat, Weibo, and Xiaohongshu. Participants voluntarily accessed and completed the survey via shared links. Before participation, all individuals were informed about the study’s purpose and provided electronic informed consent. Participants retained full autonomy throughout the process and were reminded that they could withdraw from the study at any time without penalty if they experienced any discomfort or distress.

#### 2.2.3 Measurements

##### 2.2.3.1 Socio-demographic characteristics

A self-designed demographic questionnaire was used to gather background information from participants. Variables included gender, age, education, father’s and mother’s education, residence, marital status, occupational status, family monthly income, religion, daily online time, perceptions of the current social environment, love experience, and sexual orientation.

##### 2.2.3.2 Social attitudes toward Tongqi

A self-developed questionnaire based on the ABC model of attitudes (22) was used, comprising three dimensions: cognitive (7 items), affective (8 items), and behavioral (9 items), totaling 24 items. Each item was rated on a 5-point Likert scale (1 = “strongly disagree”, 5 = “strongly agree”), with higher scores indicating more positive social attitudes. The finalized scale demonstrated excellent reliability (Cronbach’s α = 0.94). Detailed information on item pool generation, expert review, and pilot testing is provided in **Supplementary Material S1**.

##### 2.2.3.3 Marriage intention

Marriage intention was assessed using Xie JW’s Marriage Intention Scale (23), comprising 27 items across seven dimensions rated on a 7-point Likert scale. Higher scores reflect stronger marriage intentions. The scale demonstrated excellent internal consistency in this study, with a Cronbach’s α of 0.98.

##### 2.2.3.4 Online social support

The Online Social Support Scale by Nick EA (24) consist of 40 items across four dimensions, rated on a 5-point Likert scale. Higher scores indicate greater perceived online social support. In this study, the scale demonstrated excellent reliability, with a Cronbach’s α of 0.98.

##### 2.2.3.5 Homosexuality attitude

The Homosexuality Attitude Scale developed by Anderson JR (25) was translated and adapted for use in the Chinese context (**Supplementary Material S2**). It includes 16 items rated on a 7-point Likert scale; higher scores indicate more favorable attitudes toward homosexuality. The Cronbach’s α coefficient in this study was 0.82.

##### 2.2.3.6 Same-sex marriage attitudes

Attitudes toward same-sex marriage were measured using Pearl MR’s scale (26), comprising 17 items rated on a 5-point Likert scale, with higher scores indicating greater support. The Cronbach’s α coefficient for this study was 0.85.

##### 2.2.3.7 Empathy

Empathy was measured using the 20-item Perth Empathy Scale developed and validated by Brett JD (27), rated on a 5-point Likert scale. Higher scores reflect higher empathy levels. The Cronbach’s α coefficient in the current study was 0.94, indicating high internal consistency.

#### 2.2.4 Data analysis

To ensure clarity and structure in the analytical process, three hypotheses were tested in this study:

H1: There are significant differences in social attitude scores across socio-demographic characteristics.
H2: Continuous variables (e.g., marriage intention, online social support, etc.) are significantly correlated with social attitude scores.
H3: Socio-demographic and continuous variables that show significant associations in univariate or correlational analyses remain significant predictors of social attitude scores in multivariate regression analysis.

The data were analyzed by SPSS 29.0 software and Origin 2024. Prior to comparisons, the normality of all continuous variables was assessed using the Shapiro-Wilk test. Continuous variables were expressed as means ± standard deviation or median with interquartile range, while categorical variables were summarized using frequency and percentage. To better reflect the distribution of social attitudes toward Tongqi, participants were categorized into three groups according to the quartiles of the total score: a ‘Lower Score’ group (≤ P25), a ‘Medium Score’ group (P25-P75), and a ‘Higher Score’ group (≥ P75). Independent-samples *t*-tests and one-way ANOVA were conducted to compare social attitude scores across demographic groups. Pearson correlation analysis was performed to examine relationships between continuous variables. Variables that showed statistically significant (*P* < 0.05) in the univariate or correlation analyses were subsequently entered into multiple linear regression models, with the total score of the 24-item Social Attitudes Scale serving as the dependent variable. Regression assumptions were tested and satisfied, including the absence of multicollinearity (variance inflation factor < 5) and normally distributed residuals. *P* < 0.05 was defined as statistically significant.

### 2.3 Qualitative phase

#### 2.3.1 Participants

Purposive sampling was employed to recruit participants with diverse socio-demographic backgrounds. This approach aimed to capture a broad spectrum of perspectives, as variables such as educational level can significantly influence individuals’ perceptions, values, and knowledge related to the study topic. For example, participants with higher education may offer more analytical insights, while those with lower levels of formal education may provide grassroots-level perspectives, thereby enhancing the overall comprehensiveness of the study. The inclusion criteria were consistent with those used in the quantitative phase.

Sample size was determined based on the principle of data saturation, which is achieved when no new information or themes emerge from the interviews (28). To ensure the comprehensiveness of data, two additional interviews were conducted after reaching initial saturation. If no new topics emerged during these additional interviews, participant recruitment was discontinued.

#### 2.3.2 Data collection

This qualitative study was conducted through in‐depth interviews from September to December 2024. Prior to participation, all interviewees signed an informed consent form. The researcher had no prior relationship with any of the participants, thus minimizing potential bias. Interviews were conducted either face-to-face or by telephone in quiet, private settings, with no third parties present.

A preliminary semi-structured face-to-face interview guide was formulated based on a comprehensive literature review and consultations with clinicians. This guide was refined through pilot interviews with three participants to improve clarity and relevance. Sample interview questions included: (1) Do you know anything about the Tongqi community? Do you know the reasons for the creation of this group and how its lives? (2) How do you feel about Tongqi as a group? What kind of emotional attitudes do you have towards such women? (3) What do you think are some of the life struggles faced by women like Tongqi, and what kind of help do they need?

The interviews were carried out by professionally trained master’s students in nursing. Interviews followed the semi-structured guide, with flexibility to adjust question order based on participant responses. Follow-up questions, probes, and clarifications were used when necessary. All interviews were audio‐recorded with participants’ consent and transcribed verbatim within 24 hours. Transcripts were collated and returned to participants for checking, and then reviewed by two researchers for accuracy. All interviews were conducted in Chinese, later translated into English, and reviewed by all authors to ensure fidelity and consistency.

#### 2.3.3 Data analysis

Interview data were transcribed verbatim and analyzed using NVivo 14 following Colaizzi’s seven-step phenomenological analysis. Two researchers independently extracted significant statements, formulated meanings, and organized themes, which were then refined and verified through participant feedback and team discussions. This iterative process ensured conceptual accuracy and credibility. The full analytical procedure is provided in **Supplementary Material S3**.

#### 2.3.4 Rigour and reflexivity

To ensure methodological rigour and enhance reflexivity, several strategies were implemented throughout the research process. The research team, which possessed prior experience in studies involving the Tongqi community, provided a robust foundation for this study design and interpretation. To mitigate potential bias, strict recruitment protocols were enforced, prohibiting any prior interactions between researchers and participants before formal data collection commenced. A semi-structured interview guide was developed and refined based on a literature review, team discussions and pre-interviews, ensuring content relevance and comprehensiveness. All interviewers received standardized training in qualitative interviewing techniques to endure consistency and promote effective communication. Data saturation was used to confirm the adequacy of information depth and thematic breadth. Furthermore, data analysis and interpretation were conducted collaboratively by multiple researchers, enhancing the credibility, confirmability, and dependability of the findings.

### 2.4 Ethical statement

This study protocol was conducted in accordance with the ethical principles outlined in the Declaration of Helsinki. Ethical approval was obtained from the Ethics Committee of the Nursing College at Hubei University of Chinese Medicine. All participants were fully informed about the purpose, procedures, and potential risks of the study, and each provided written informed consent prior to participation. Written informed consent was obtained from the participants for the publication of any potentially identifiable data included in this article.

## 3 Results

### 3.1 Quantitative study

#### 3.1.1 Participants’ characteristics

A total of 650 questionnaires were distributed, with 587 valid responses returned, resulting in an effective recovery rate of 90.31%. Unanswered questionnaires were excluded from the analysis. As shown in **Table 1**, among the 587 participants, 279 (47.53%) were female, and 308 (52.47%) were male. Regarding love experience, the majority of participants (n = 464, 79.05%) reported having love experience. The sample was predominantly well-educated, with 437 participants (74.45%) holding at least a bachelor’s degree. Furthermore, the majority identified as heterosexual (502, 85.52%).

**Table 1:** Socio-demographic characteristics of the participants (N = 587).

Groups	Number	Percent	Groups	Number	Percent
Gender			Age (years)		
Male	308	52.47	18 ~ 30	330	56.21
Female	279	47.53	≥ 31	257	43.79
Education			Love experience		
Below bachelor’s degree	150	25.55	Yes	464	79.05
Bachelor’s degree or above	437	74.45	No	123	20.95
Father’s education			Mother’s education		
Junior high school or below	215	36.63	Junior high school or below	233	39.69
High school	170	28.96	High school	170	28.96
College or above	202	34.41	College or above	184	31.35
Marital status			Occupational status		
Unmarried	239	40.72	Employed	308	52.47
In romantic relationships	170	28.96	Unemployed	279	47.53
Married	178	30.32			
Family monthly income			Residence		
< 3000	123	20.95	Tier 1 or 2 cities	156	26.58
3000 ~ 4999	156	26.58	Tire 3 cities	168	28.62
5000 ~ 10000	187	31.86	Small cities	151	25.72
> 10000	121	20.61	Townships or rural areas	112	19.08
Religion			Daily online time		
Yes	81	13.79	< 6 hours/day	320	54.51
No	506	86.21	≥ 6 hours/day	267	45.49
Social environment			Sexual orientation		
Patriarchal/Matriarchal society	200	34.07	Heterosexual	502	85.52
Gender-equal society	387	65.93	Heterosexual/Bisexual	85	14.48

#### 3.1.2 The scores of the general public’s social attitudes toward Tongqi

The social attitudes toward Tongqi yielded a total score of 89.93 ± 16.60. The scores for the cognitive, affective, and behavioral dimensions were 24.85 ± 5.03, 29.40 ± 6.21, and 35.67 ± 7.14, respectively. As detailed in **Table 2**, the total score distribution (P25 = 80, P50 = 92, P75 = 102) informed the categorization of participants into Lower, Medium, and Higher Score groups for subsequent analysis.

**Table 2:** Score of social attitudes toward Tongqi among the general public.

	N	Min	Max	Total score (Mean ± SD)	Item score (Mean ± SD)	P25	P50	P75
Social attitudes	587	24	120	89.93 ± 16.60	3.75 ± 0.69	80	92	102
Cognitive attitude	587	7	35	24.85 ± 5.03	3.55 ± 0.72	21	25	28
Affective attitude	587	8	40	29.40 ± 6.21	3.68 ± 0.78	25	30	34
Behavioral attitude	587	9	45	35.67 ± 7.14	3.96 ± 0.79	31	36	40

#### 3.1.3 Factors related to the general public’s social attitudes toward Tongqi

##### 3.1.3.1 H1: Differences across socio-demographic characteristics

To test H1, univariate analyses were conducted to explore differences in social attitudes scores across socio-demographic characteristics. Several factors were significantly associated with social attitudes, including gender, education, father’s education, mother’s education, marital status, occupational status, religion, perceptions of the current social environment, love experience, and sexual orientation (all *P* < 0.01). Detailed results are presented in **Table 3**.

**Table 3:** Univariate analysis of social attitudes toward Tongqi among the general public with different demographic characteristics (N = 587).

	Social attitudes			Cognitive attitude			Affective attitude			Behavioral attitude		
	Score	t/F	*P*	Score	t/F	*P*	Score	t/F	*P*	Score	t/F	*P*
Gender												
Male Female	3.63 ± 0.67 3.87 ± 0.69	-4.44	<0.001	3.53 ± 0.70 3.57 ± 0.74	-0.75	-0.46	3.56 ± 0.77 3.80 ± 0.77	-3.72	<0.001	3.76 ± 0.74 4.19 ± 0.79	-6.69	<0.001
Education												
Below bachelor degree Bachelor’s degree or above	3.50 ± 0.62 3.83 ± 0.70	-5.00	<0.001	3.41 ± 0.63 3.60 ± 0.74	-2.87	<0.01	3.48 ± 0.68 3.74 ± 0.80	-3.67	<0.001	3.62 ± 0.69 4.08 ± 0.79	-6.44	<0.001
Father’s education												
Junior high school or below High school College or above	3.81 ± 0.62 3.65 ± 0.74 3.89 ± 0.68	4.95	<0.01	3.52 ± 0.65 3.51 ± 0.74 3.77 ± 0.77	4.57	<0.05	3.75 ± 0.69 3.58 ± 0.83 3.81 ± 0.76	4.32	<0.05	4.09 ± 0.74 3.85 ± 0.83 4.04 ± 0.71	6.58	<0.01
Mother’s education												
Junior high school or below High school College or above	3.81 ± 0.61 3.67 ± 0.75 3.84 ± 0.64	3.71	<0.05	3.52 ± 0.66 3.54 ± 0.77 3.69 ± 0.70	1.41	0.25	3.74 ± 0.69 3.59 ± 0.85 3.80 ± 0.72	3.24	<0.05	4.09 ± 0.74 3.85 ± 0.83 4.04 ± 0.71	7.87	<0.001
Marital status												
Unmarried In romantic relationships Married	3.91 ± 0.64 3.63 ± 0.65 3.65 ± 0.75	11.38	<0.001	3.62 ± 0.69 3.40 ± 0.68 3.59 ± 0.78	4.96	<0.01	3.84 ± 0.72 3.54 ± 0.75 3.59 ± 0.85	9.06	<0.001	4.19 ± 0.76 3.87 ± 0.76 3.74 ± 0.79	19.20	<0.001
Occupational status												
Employed Unemployed	3.62 ± 0.73 3.88 ± 0.62	-4.69	<0.001	3.53 ± 0.74 3.57 ± 0.69	-0.70	0.49	3.54 ± 0.83 3.83 ± 0.68	-4.57	<0.001	3.77 ± 0.79 4.18 ± 0.73	-6.51	<0.001
Religion												
Yes No	3.77 ± 0.70 3.58 ± 0.61	2.29	<0.05	3.57 ± 0.73 3.45 ± 0.63	1.49	0.14	3.70 ± 0.78 3.51 ± 0.74	2.04	<0.05	4.00 ± 0.81 3.75 ± 0.67	2.97	<0.01
Social environment												
Patriarchal/Matriarchal society Gender-equal society	3.85 ± 0.66 3.69 ± 0.70	2.67	<0.01	3.66 ± 0.70 3.49 ± 0.72	2.68	<0.01	3.79 ± 0.69 3.62 ± 0.81	2.79	<0.01	4.06 ± 0.73 3.92 ± 0.82	2.02	<0.05
Sexual orientation												
Heterosexual Heterosexual/Bisexual	3.71 ± 0.71 3.91 ± 0.53	-2.41	<0.05	3.52 ± 0.75 3.74 ± 0.47	-3.58	<0.001	3.64 ± 0.80 3.88 ± 0.57	-3.45	<0.001	3.94 ± 0.81 4.07 ± 0.68	-1.40	0.16
Love experience												
Yes No	3.71 ± 0.71 3.88 ± 0.59	-2.74	<0.05	3.54 ± 0.74 3.60 ± 0.64	-0.99	0.32	3.64 ± 0.80 3.83 ± 0.66	-2.75	<0.01	3.91 ± 0.80 4.15 ± 0.72	-2.95	<0.01

##### 3.1.3.2 H2: Correlations with continuous variables

**Figure 1:**
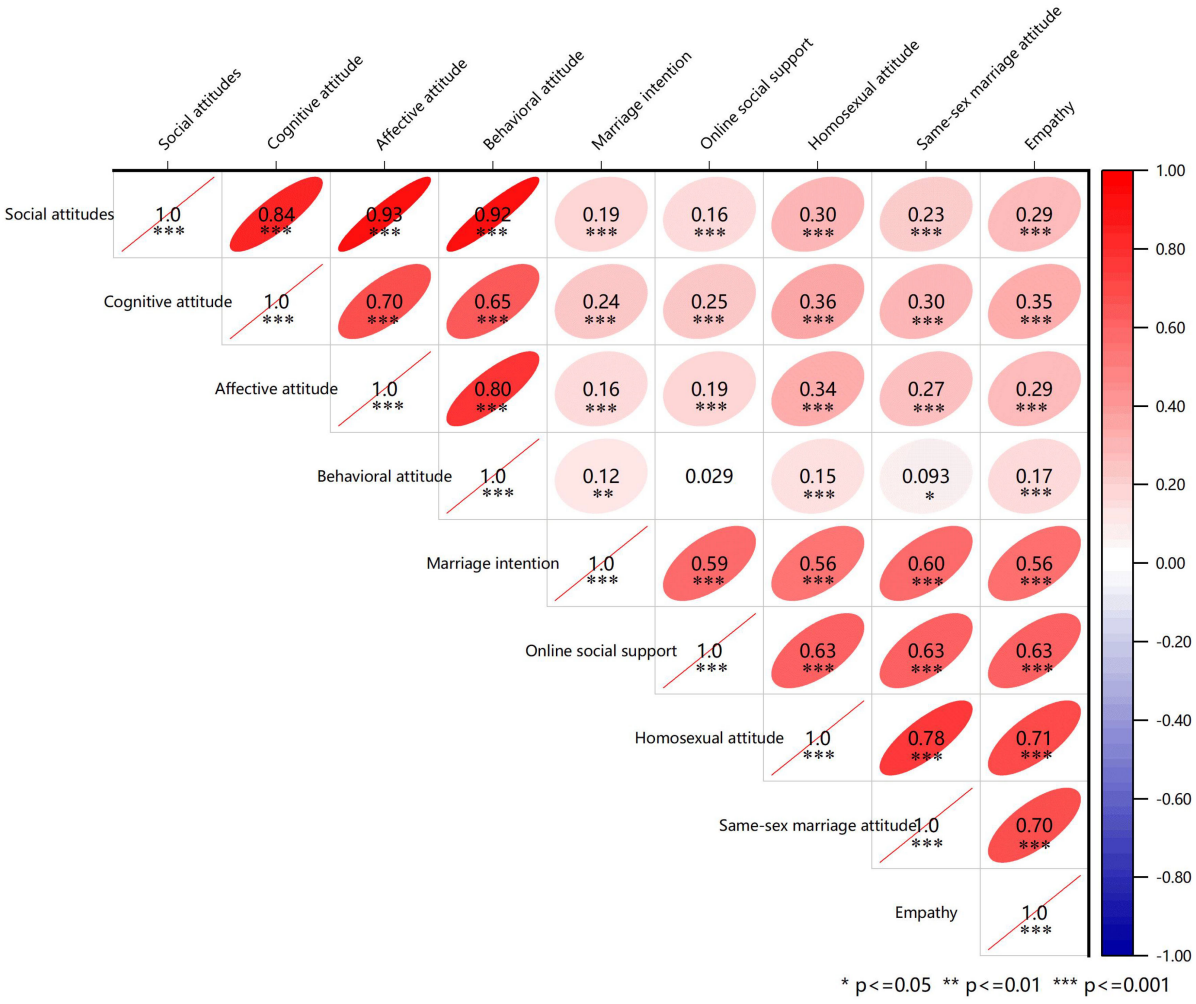
Pearson correlation matrix between the variables: the heatmap compares the Pearson correlation coefficients and the degree of correlation among the variables. Each cell in the heatmap represents the Pearson correlation coefficient between two variables, with the color intensity and the value indicating the strength and direction of the correlation. Asterisks indicates significant values: *p < 0.05, **p < 0.01, ***p < 0.001.

To test H2, Pearson correlation analyses were performed to examine relationships between continuous variables and social attitude scores. Social attitudes were positively correlated with marriage intention (*r* = 0.19, *P* < 0.001), online social support (*r* = 0.16, *P* < 0.001), homosexual attitude (*r* = 0.30, *P* < 0.001), same-sex marriage attitude (*r* = 0.23, *P* < 0.001), and empathy (*r* = 0.29, *P* < 0.001). Additionally, the cognitive, affective and behavioral attitude dimensions were moderately positively correlated with each other (*r* = 0.65-0.80, *P* < 0.05). **Figure 1** presents the detailed results.

##### 3.1.3.3 H3: Multivariate predictors of social attitudes

To test H3, multiple linear regression analyses were conducted with social attitude scores as the dependent variable, including all variables that were significant in the univariate and correlation analyses as independent variables. The regression model was statistically significant (*F* = 15.44, *P* < 0.001), and nine variables were retained in the final regression equation (**Table 4**). Among these, gender, education, occupational status, sexual orientation were positively associated with social attitudes, whereas marital status and perceptions of the current social environment were negatively associated.

**Table 4:** Multivariate analysis of the general public’s social attitudes toward Tongqi.

Dependent variable	Independent variable	*β*	SE	*β’*	t	*P*
Social attitudes	Constant	23.95	8.07		2.97	<0.01
	Gender	5.39	1.59	0.16	3.38	<0.001
	Education	3.37	1.65	0.09	2.05	<0.05
	Marital status	-3.45	1.04	-0.17	-3.31	<0.001
	Occupation status	3.99	1.81	0.12	2.20	<0.05
	Social environment	-4.83	1.34	-0.14	-3.62	<0.001
	Sexual orientation	5.66	1.82	0.12	3.11	<0.01
	Marriage intention	0.16	0.04	0.22	4.29	<0.001
	Homosexual attitude	0.28	0.08	0.21	3.42	<0.001
	Empathy	0.16	0.07	0.12	2.16	<0.05
Cognitive attitude	Constant	8.16	1.98		4.11	<0.001
	Education	1.33	0.47	0.12	2.86	<0.01
	Marital status	-1.14	0.29	-0.19	-3.96	<0.001
	Social environment	-1.07	0.41	-0.10	-2.60	<0.01
	Sexual orientation	1.39	0.56	0.10	2.49	<0.05
	Marriage intention	0.03	0.01	0.13	2.47	<0.05
	Homosexual attitude	0.09	0.03	0.23	3.51	<0.001
	Empathy	0.05	0.02	0.14	2.31	<0.05
Affective attitude	Constant	4.49	3.03		1.48	0.14
	Gender	1.59	0.60	0.13	2.65	<0.01
	Marital status	-1.16	0.39	-0.16	-2.97	<0.01
	Occupation status	2.14	0.68	0.17	3.14	<0.01
	Social environment	-1.48	0.50	-0.11	-2.95	<0.01
	Sexual orientation	2.03	0.68	0.12	2.96	<0.01
	Marriage intention	0.04	0.01	0.13	2.52	<0.05
	Homosexual attitude	0.13	0.03	0.26	4.16	<0.001
	Same-sex marriage attitude	0.09	0.04	0.14	2.15	<0.05
Behavioral attitude	Constant	14.33	3.43		4.18	<0.001
	Gender	2.75	0.70	0.19	3.92	<0.001
	Education	1.86	0.73	0.11	2.57	<0.01
	Marital status	-1.53	0.46	-0.18	-3.33	<0.001
	Occupation status	1.75	0.80	0.12	2.20	<0.05
	Social environment	-2.23	0.58	-0.15	-3.84	<0.001
	Marriage intention	0.08	0.02	0.25	4.83	<0.001

### 3.2 Qualitative study

In the qualitative phase of the study, various dimensions of social attitudes, including cognitive attitude, affective attitude and behavioral attitude, were obtained. The researchers interviewed twenty participants to explore the general public’s social attitudes toward Tongqi. The participants were indicated separately as P1-P20, ranging between 20–40 years of age (median = 28 years). **Table 5** lists the individual characteristics of the participants.

**Table 5:** Interview information and characteristics of the participants.

Code	Age-ranges	Gender	Education	Careers	Marital status	Interview format	Sexual orientation	Homosexuality attitudes
P1	<30	Female	Undergraduate	Economics	Unmarried	Telephone	Bisexual	Approve
P2	<30	Male	Undergraduate	Policeman	Unmarried	Telephone	Heterosexual	Opposed
P3	<30	Female	Bachelor’s degree	Student	Unmarried	Face-to-face	Heterosexual	Approve
P4	≥30	Male	Junior High School	Self-employed	Married	Telephone	Heterosexual	Opposed
P5	<30	Male	Bachelor’s degree	Student	Unmarried	Face-to-face	Heterosexual	Opposed
P6	<30	Female	Undergraduate	Nurse	Unmarried	Telephone	Heterosexual	Approve
P7	<30	Female	Bachelor’s degree	Student	Unmarried	Face-to-face	Heterosexual	Opposed
P8	<30	Female	Bachelor’s degree	Student	Unmarried	Face-to-face	Heterosexual	Opposed
P9	<30	Male	Undergraduate	NA	Unmarried	Telephone	Heterosexual	Opposed
P10	<30	Female	Bachelor’s degree	Student	Unmarried	Face-to-face	Heterosexual	Approve
P11	<30	Female	Undergraduate	NA	Unmarried	Telephone	Heterosexual	Approve
P12	<30	Male	Bachelor’s degree	Student	Unmarried	Telephone	Heterosexual	Opposed
P13	<30	Female	Bachelor’s degree	Student	Unmarried	Face-to-face	Heterosexual	Opposed
P14	≥30	Male	Bachelor’s degree	Doctor	Married	Face-to-face	Heterosexual	Opposed
P15	<30	Male	Undergraduate	Student	Unmarried	Telephone	Heterosexual	Opposed
P16	<30	Male	Undergraduate	Economics	Unmarried	Telephone	Heterosexual	Opposed
P17	<30	Female	Bachelor’s degree	Nurse	Unmarried	Face-to-face	Heterosexual	Opposed
P18	<30	Male	Bachelor’s degree	Engineering	Unmarried	Telephone	Heterosexual	Approve
P19	<30	Female	Undergraduate	Nurse	Unmarried	Telephone	Heterosexual	Approve
P20	<30	Male	Undergraduate	Nurse	Unmarried	Telephone	Heterosexual	Opposed

At this stage, eight subcategories and three main themes were identified as the consequences of social attitudes toward Tongqi among the general public. The main themes included manifestation under the restraint of traditional Chinese culture, an impulse to embrace, and a need for multifaceted support (**Table 6**).

**Table 6:** Social attitudes of the general public toward Tongqi (themes and sub-themes).

Dimension	Themes	Sub-themes
Cognitive aspect	Awareness shaped by traditional and societal norms	Influence of traditional Chinese cultural values
		Perceived absence of social justice
Emotional aspect	Public empathy and emotional orientation toward Tongqi	Respect and moral support
		Inclusion and emotional understanding
Behavioral aspect	Expressed needs and public expectations for support mechanisms	Need for public education
		Call for broader social acceptance
		Psychological support needs
		Legal protection and advocacy

#### 3.2.1 Theme 1: Awareness shaped by traditional and societal norms—— cognitive aspect

The “Tongqi” phenomenon is closely linked to deep-rooted traditional Chinese cultural values concerning marriage, family, and sexual orientation. These norms uphold heterosexual marriage as the primary means for ensuring lineage continuity and cultural inheritance, exerting significant pressure on homosexual individuals to conform to conventional marital expectations. Consequently, the existence of Tongqi reflects a unique manifestation of traditional ideology within a specific social context and highlights the cognitive tension between enduring beliefs and evolving modern views on gender and sexuality. This phenomenon further illustrates the slow pace of social change and the enduring influence of traditional norms on individual perceptions and marital choices.

##### 3.2.1.1 Sub-Theme 1: Influence of traditional Chinese cultural values

Traditional Chinese culture, rooted in filial piety and Confucian ideals, places paramount importance on procreation and continuation of the family line. Concepts such as *“Among the three forms of unfilial conduct, the worst is to have no descendants”* have long shaped societal expectations around marriage. Within this framework, disclosing one’s homosexual orientation is often regarded as a betrayal of family duty, prompting many individuals to enter heterosexual marriages to satisfy cultural and familial norms. Thus, the Tongqi phenomenon exemplifies the internal conflict between cultural expectations and personal identity.

“I think there is no way to be recognized by society because this concept is still a little ahead of its time. In China, whether from the perspective of morality or humanism, the existence of this group is still quite controversial.” (P11).

“That may be forced by family reasons, like parents asking you to get married. Traditional Chinese culture emphasizes continuing the family line—it’s a typical expectation in Chinese marriage culture.” (P15).

“People are still constrained by traditional marriage concepts, the need to pass on the family name, and pressure from parents and society.” (P18).

These accounts collectively revealed how deeply entrenched cultural norms of filial piety and lineage preservation continue to govern individual marital choices. They demonstrated that social conformity often overrides personal identity, reflecting the cognitive dissonance faced by homosexual individuals caught between traditional obligations and evolving modern values. This also illustrated how moral and familial expectations perpetuate the persistence of the Tongqi phenomenon within contemporary Chinese society.

##### 3.2.1.2 Sub-Theme 2: Perceived absence of social justice

Owing to their spouses’ concealed sexual orientation, many Tongqi find themselves in asymmetrical and emotionally unfulfilling marital relationships. They often endure emotional neglect, psychological harm, and in some cases, covert domestic abuse, with limited legal recourse. At the societal level, protection mechanisms are underdeveloped, and legal frameworks lag in addressing their specific needs, particularly regarding divorce, child custody, and property rights. This lack of legal and institutional support underscores a broader perception of structural injustice faced by Tongqi.

“Of course, it is unfair. Women are the most vulnerable. Most people don’t understand this group, and there’s no legal protection.” (P2).

“It deprives women of the opportunity to build a normal family. This behavior is essentially a form of deception.” (P5).

“Women can be easily deceived … They bear psychological burdens and are left emotionally and physically unfulfilled.” (P17).

These accounts highlighted how the lack of institutional safeguards and persistent gender inequalities contribute to a perceived sense of social injustice among Tongqi. The narratives illustrated both the emotional consequences of deception and the systemic failure to protect women’s rights, reinforcing public perceptions of moral imbalance and legal insufficiency within current marital structures.

#### 3.2.2 Theme 2: Public empathy and emotional orientation toward Tongqi —— emotional aspect

Public attitudes exhibit a growing emotional awareness characterized by empathy, moral support, and inclusion. The public increasingly recognizes the challenges and injustices Tongqi face and affirms their dignity and value. Respect implies treating them as equal social actors; support involves both emotional solidarity and practical advocacy; inclusion reflects a willingness to abandon prejudice and understand their lived experiences. These emotional responses signal a shift toward greater social awareness and ethical responsibility.

##### 3.2.2.1 Sub-Theme 1: Respect and moral support

Respect entails acknowledging Tongqi as autonomous individuals with inherent dignity, rather than reducing them to their marital roles. Moral support includes encouraging them to confront difficulties, make independent decisions, and seek life changes. Moreover, public discourse and media advocacy can serve as vehicles for visibility and empowerment.

“I respect that. I respect Tongqi’s wishes.” (P5).

“They may not know whether their husbands married them for love or to hide their sexual orientation.” (P10).

“We should respect their autonomy, including decisions like staying in the marriage—it’s about honoring personal choice.” (P16).

These statements indicated that respect for Tongqi is evolving from pity toward a recognition of personal agency. Participants emphasized that acknowledging Tongqi’s right to self-determination represents a critical move toward social equality. This moral respect underscored the growing ethical maturity of public attitudes, positioning Tongqi as subjects of empowerment rather than victims of circumstance.

##### 3.2.2.2 Sub-Theme 2: Inclusion and emotional understanding

Public empathy toward Tongqi reflects a gradual shift from stigmatization to recognition. Many individuals have begun to appreciate the emotional distress, loneliness, and moral dilemma experienced by Tongqi. This empathetic stance fosters social acceptance and lays a foundation for psychological reassurance and community integration.

“At first, I felt uneasy, but now I can understand and accept their existence.”(P11).

“In general, people are more accepting now because they’ve been treated unfairly.” (P14).

“Promoting tolerance can reduce divisions and create a friendlier environment that respects diversity.” (P16).

These quotations collectively indicated a softening of social attitudes toward Tongqi, signaling a transition from judgment to empathy. They reflected a broader societal evolution in which emotional understanding serves as a precursor to inclusion. Such empathy not only reduced stigma but also facilitated dialogue and promoted the normalization of diverse marital experiences.

#### 3.2.3 Theme 3: Expressed needs and public expectations for support mechanisms

Due to stigma and marital vulnerability, many Tongqi are reluctant to seek help or reveal their identities. Public understanding remains limited, yet awareness is growing about the multifaceted support Tongqi need. Participants identified four primary areas of concern: public education, social acceptance, psychological services, and legal protections. These needs reflect not only the behavioral challenges faced by Tongqi but also a public expectation for systemic response and social reform.

##### 3.2.3.1 Sub-theme 1: need for public education

Misconceptions about Tongqi often stem from limited public knowledge regarding homosexuality and non-normative marriages. Participants emphasized the critical role of integrated educational initiatives, including school-based programs, media outreach, and community workshops, in promoting a more informed and tolerant society.

“We should educate women about their rights so they can better advocate for themselves.” (P1).

“Public education is key to making society more inclusive of LGBTQ individuals.”(P7).

“Improving sex education will help teenagers understand sexual orientation more clearly.” (P16).

“There should be ways to help women recognize whether a man is gay—this could prevent such tragedies.” (P17).

These views collectively underscored education as the foundation for changing public perceptions and reducing prejudice. Participants recognized that early and comprehensive education can bridge informational gaps, challenge heteronormative assumptions, and foster societal empathy toward both Tongqi and LGBTQ individuals.

##### 3.2.3.2 Sub-theme 2: call for broader social acceptance

Participants called for action-oriented inclusion of Tongqi in everyday social life. Equal treatment in the workplace, positive representation in public discourse, and visibility in mainstream media were considered essential. Such strategies would help reduce stigma and normalize the existence of Tongqi.

“Society should enhance campaigns to raise awareness of these groups.” (P7).

“We need to help people understand the origins of this group and promote acceptance.”(P9).

“Even if not everyone accepts them, at least they won’t be viewed with discrimination.” (P11).

“Encouraging openness can help reduce misunderstanding and bias.” (P20).

These comments highlighted the growing recognition that inclusion must extend beyond sympathy to tangible social participation. By advocating for equal visibility and representation, participants framed acceptance as an ethical and civic responsibility, essential for dismantling stigma and promoting social harmony.

##### 3.2.3.3 Sub-theme 3: psychological support needs

Many Tongqi endure significant psychological distress due to betrayal, emotional deprivation, and social alienation. Participants advocated for targeted mental health services, including access to professional counseling, emotional support systems, and safe spaces for self-expression.

“Psychological counseling can help them fill emotional gaps through family or friendships.” (P3).

“There should be dedicated counseling services for this group.” (P11).

“Psychological support can help them come to terms with their situation.” (P12).

“Trauma and discrimination make psychological support even more important.” (P13).

These quotations revealed a widespread recognition of the psychological toll experienced by Tongqi. The emphasis on counseling and emotional support reflected an emerging societal understanding of mental health as a public concern, underscoring the need for professional, gender-sensitive services to promote recovery and resilience.

##### 3.2.3.4 Sub-theme 4: legal protection and advocacy

Tongqi often faces legal disadvantages in marital disputes. Participants stressed the importance of specific legal reforms, improved access to legal aid, and increased public legal literacy. Robust legal frameworks would ensure that Tongqi are better protected and afforded equal treatment.

“There is also legislation. For example, the National People’s Congress can enact some relevant legislation or make some amendments to the law, and local governments in various places can also adjust their systems accordingly to safeguard the rights of the homosexual community.” (P5).

“I think that for women with divorce claims, I think that this marriage law is not able to introduce some policies for special groups, that is, the direction of the law, and then go a little bit toward the Tongqi group.” (P10).

“Then there’s legal aid, I guess, and there’s a need for legal aid in this area because a lot of guys are just out to cheat on their marriage and have babies, and women need to be given more help and rights in the eyes of the law.” (P13).

“For this kind of marriage problem, I think the state should introduce some laws to help them.” (P15).

These accounts collectively emphasized the urgent need for structural and legal interventions. Participants viewed legislative reform and accessible legal aid as not merely protective measures but as indicators of societal recognition and justice. Such perspectives highlighted the role of institutional change in achieving gender equity and safeguarding marital rights for vulnerable groups.

## 4 Discussion

To our knowledge, this was the first mixed-methods study in China to systematically examine public attitudes toward Tongqi. By integrating quantitative and qualitative approaches, it provided a comprehensive analysis of the cognitive, affective, and behavioral dimensions of social attitudes, as well as the influencing sociodemographic and perceptual factors. Unlike previous research that primarily focused on the lived experiences of Tongqi, this study shifted the lens to the broader public, thereby offering a more objective understanding of how this marginalized group is perceived within the prevailing sociocultural context. The findings enriched the existing literature on public attitudes toward marginalized groups and offered practical implications for developing strategies to foster greater social inclusion and reduce stigma in contemporary Chinese society.

The overall neutral public attitudes toward Tongqi reflected a complex interplay between individual sociodemographic characteristics and deeply rooted sociocultural norms surrounding gender, sexuality, and marriage in China. Quantitatively, factors such as gender, education, marital status, occupational status, perception of the current social environment, and sexual orientation may be significant predictors. Female respondents and those with higher education levels exhibited more positive attitudes, a pattern consistent with prior studies (29, 30). A study in a mid-sized Canadian university similarly found that women, individuals with progressive education, and those with direct contact with sexual minorities held more favorable attitudes, whereas men, politically conservative, and religious individuals tended to express more negative or neutral views (31). Qualitative interviews provided a deeper understanding of these patterns. Female and higher-educated participants often reported greater exposure to diverse perspectives, more critical reflections on traditional gender norms, and higher empathy toward marginalized groups. Many expressed awareness of the moral and familial pressures faced by Tongqi. Unmarried individuals also held more positive social attitudes toward Tongqi, potentially due to greater openness regarding marriage and sexual orientation, critical thinking about traditional social norms, and empathy for the spouses of gay men (32). Qualitative narratives supported this, showing that unmarried participants frequently reflected on societal expectations and empathized with Tongqi’s constrained choices. Furthermore, individuals who perceived the current social environment as unequal may be more sympathetic toward Tongqi, likely due to their sensitivity to gender inequality, commitment to social justice, and critique of traditional gender roles (5, 6). Similarly, respondents identifying as gay or bisexual reported significantly more favorable attitudes, which may stem from shared experiences of discrimination, greater identification with sexual minority struggles, and a stronger inclination to support the rights of affected populations (5, 33). These patterns were echoed in qualitative findings, where participants described awareness of social stigma, empathy for marginalized experiences, and recognition of systemic injustices faced by Tongqi. This integrative interpretation demonstrateed how personal perception interacts with broader structural and cultural factors to shape public attitudes.

Qualitative findings revealed a notable cognitive-emotional-behavioral gap in public perceptions. While many participants expressed emotional sympathy toward Tongqi, such affective responses rarely translated into supportive actions. Cognitively, public awareness of Tongqi remains limited and fragmented. Most interviewees attributed the phenomenon to the low social acceptance of homosexuality, which forces some gay men into heterosexual marriages as a form of concealment. Consistent with prior research, social stigma and fear of discrimination often compelled Tongqi to remain silent and invisible (34). Beyond societal marginalization, Tongqi also faces intense moral and familial pressure to maintain traditional marital roles to preserve family reputation and fulfill child-rearing responsibilities (2, 35–37). Emotionally, many respondents expressed sympathy and respect, recognizing that most Tongqi do not voluntarily choose such marital arrangements but rather succumb to cultural expectations and age-related marriage pressures (2, 37). Behaviorally, however, tangible support remains scarce. Participants frequently emphasized the urgent need for comprehensive sexual education, broader public acceptance of sexual minorities, psychological counseling services, and accessible legal assistance for Tongqi. The lack of sex education not only fosters misunderstandings about homosexuality, such as the belief that it can be “corrected,” but also contributes to unsafe sexual practices (1, 17). These conditions elevate HIV transmission risks (38), exacerbated by the high prevalence of unprotected sex and the limited sexual autonomy among Tongqi. Existing studies reported that 9.70% of Tongqi engage in extramarital sexual relationships, further compounding HIV-related risks (9, 17). The integration of quantitative and qualitative findings provided a more comprehensive understanding of these dynamics. The quantitative neutral attitudes observed quantitatively aligned with qualitative accounts of emotional sympathy coupled with limited behavioral support. The qualitative data elucidated why certain sociodemographic groups (e.g., women, higher-educated, unmarried, LGBTQ) express greater empathy, how entrenched societal norms inhibit supportive actions, and what structural gaps prevent sympathy from translating into concrete support.

The findings highlighted the critical need to foster a more supportive social climate toward Tongqi. Interventions should be informed by quantitative trends in individual attitudes and qualitative insights into the broader societal context and support mechanisms. Bridging the identified cognitive-affective-behavioral gap requires multi-level strategies. Enhancing inclusive cultural norms, strengthening empathy through education and media, and providing clear behavioral pathways for supportive action are essential. In practice, bolstering formal and community-based sex education could help correct misconceptions and foster empathy. Community organizations and media platforms are pivotal in promoting dialogue, visibility, and behavioral engagement. Furthermore, establishing robust legal and institutional support mechanisms, including counseling services, anti-discrimination measures, and accessible legal aid, would offer tangible protection for Tongqi. Importantly, these findings carry direct implications for mental health policy development. Policy makers could leverage these insights to design targeted interventions addressing the psychological, social, and legal needs of Tongqi individuals. Examples include implementing specialized mental health counseling programs, integrating comprehensive sexual and relational education into school curricula, promoting public campaigns to reduce stigma, and strengthening legislative protections for sexual minorities. Such policy-driven initiatives can help ensure that individual empathy and awareness observed at the personal level are translated into systematic support, thereby improving the mental health and overall well-being of Tongqi populations.

## 5 Limitations

This study has certain limitations. The sample, though diverse within Hubei Province, it relied on a highly homogeneous group, with an over-representation of highly educated and unmarried individuals. Consequently, the findings may primarily reflect the attitudes of this specific subgroup rather than the broader Chinese population, limiting external validity. Moreover, urban-rural differences and regional cultural variations within Hubei may influence public attitudes toward Tongqi, and these factors were not systematically analyzed in the current study. The qualitative component also lacked gender stratification, which may have limited comprehensive insights into gender-specific views. Future research should consider expanding the sample size, geographic scope, and demographic diversity to improve representativeness. In addition, while the self-developed questionnaire may carry some self-report bias, it was rigorously tested for reliability and validity, and designed to reflect diverse age perspectives. These limitations notwithstanding, the mixed-methods design enhances the credibility and depth of the findings.

## 6 Conclusions

This mixed-methods study revealed that public attitudes toward Tongqi in Hubei Province are moderately neutral and influenced by factors such as gender, education, marital and occupational status, sexual orientation, and perceptions of the social environment. Although some cognitive awareness and emotional sympathy exist, the absence of comprehensive understanding and structural support impedes meaningful behavioral responses. The qualitative findings further exposed deep-rooted stigma and limited public knowledge, underscoring the need for targeted education and advocacy. Enhancing public sex education, reducing discrimination, and establishing multi-level support systems are essential to improving the living conditions of Tongqi and promoting social inclusion. These results emphasized that multisectoral efforts are not only for shifting societal perceptions but also for charting practical directions for future research and policy development.

## Data Availability

The raw data supporting the conclusions of this article will be made available by the authors, without undue reservation.
