# Supplementary material for: Social attitudes toward Tongqi among the general public and associated determinants: a mixed-methods study in Hubei Province, China
Source: Front Psychiatry. 2025 Nov 21;16:1700396. doi: 10.3389/fpsyt.2025.1700396 (PMC12679579; doi:10.3389/fpsyt.2025.1700396)
Supplement: Supplementary file 1 [file DataSheet1.pdf]

Supplementary Material S1: Development of the Social Attitudes toward Tongqi Scale

The Social Attitudes toward Tongqi Scale was developed following the ABC model of attitudes, which conceptualizes attitudes as consisting of cognitive, affective, and behavioral components. The development process consisted of three stages:

Stage 1: Item generation

An initial pool of 35 items was generated through a comprehensive literature review on social attitudes toward marginalized groups, gender and sexuality studies, and marriage-related stigma. In addition, semi-structured interviews were conducted with 20 adults (aged 19–60 years) from diverse educational and occupational backgrounds to capture culturally grounded perspectives. After merging overlapping items and removing redundant items, 24 items reflecting the three attitudinal components (7 cognitive, 8 affective, and 9 behavioral) were retained.

Stage 2: Expert content validation

A panel of 15 experts in psychology, sociology, and social research evaluated each item for clarity, relevance, and representativeness using a 4-point rating scale. The item-level content validity index (I-CVI) ranged from 0.87 to 1.00, and the scale-level CVI (S-CVI) was 0.94, indicating strong content validity. Feedback from experts led to minor revisions to improve linguistic precision and conceptual clarity.

Stage 3: Pilot testing

A pilot study was conducted among 50 participants from the target population to assess item comprehensibility and feasibility. Internal consistency reliability (Cronbach's $\alpha = 0.91$) and corrected item–total correlations supported the adequacy of the scale structure. No major wording issues were reported. The finalized version included 24 items and was used in the main study (Cronbach's $\alpha = 0.94$).
