# Supplementary material for: Social attitudes toward Tongqi among the general public and associated determinants: a mixed-methods study in Hubei Province, China
Source: Front Psychiatry. 2025 Nov 21;16:1700396. doi: 10.3389/fpsyt.2025.1700396 (PMC12679579; doi:10.3389/fpsyt.2025.1700396)
Supplement: Supplementary file 2 [file DataSheet2.pdf]

Supplementary Material S2: Cultural Adaptation and Reliability Testing of the Homosexuality Attitude Scale

The Homosexuality Attitude Scale (Anderson JR, 2018) was translated and culturally adapted for use in the Chinese context following international guidelines for cross-cultural adaptation of instruments. The major process was conducted in three stages：

Stage 1: Translation and back-translation

Two bilingual experts independently translated the original English version into Chinese. The translations were reconciled into a single draft and back-translated by two other bilingual experts blinded to the original version. Discrepancies were reviewed and resolved by an expert committee to ensure semantic and conceptual equivalence.

Stage 2: Cultural adaptation

Ten participants from different educational backgrounds completed the pre-final questionnaire version to assess item clarity, relevance, and cultural appropriateness. Minor linguistic modifications were made for readability and cultural sensitivity.

Stage 3: Reliability testing

A pilot test with 80 participants demonstrated acceptable internal consistency (Cronbach's $\alpha = 0.82$). Exploratory factor analysis confirmed the unidimensional structure consistent with the original scale. The final Chinese version was used in the present study.
