# Supplementary material for: Social attitudes toward Tongqi among the general public and associated determinants: a mixed-methods study in Hubei Province, China
Source: Front Psychiatry. 2025 Nov 21;16:1700396. doi: 10.3389/fpsyt.2025.1700396 (PMC12679579; doi:10.3389/fpsyt.2025.1700396)
Supplement: Supplementary file 3 [file DataSheet3.pdf]

Supplementary Material S3: Colaizzi's Seven-Step Phenomenological Analysis Procedure

The qualitative data obtained from semi-structured interviews were analyzed using Colaizzi's seven-step phenomenological method. The steps were conducted as follows:

1. Familiarization: Read all transcripts multiple times to obtain a general sense of participants' experiences.

2. Significant statement extraction: Extract statements directly related to perceptions, feelings, and experiences.

3. Formulation of meanings: Interpret significant statements to generate formulated meanings.

4. Organization into theme clusters: Group formulated meanings into thematic clusters reflecting broader conceptual patterns.

5. Exhaustive description: Synthesize themes into a comprehensive description of the phenomenon.

6. Fundamental structure identification: Derive essential structure of the experience to capture core meanings.

7. Validation: Participants reviewed and confirmed findings for accuracy and authenticity.

All analyses were conducted independently by two researchers and verified through team discussions to ensure credibility, confirmability, and dependability.
